# Two new species of *Otoba* (Myristicaceae) from Colombia

**DOI:** 10.3897/phytokeys.178.64564

**Published:** 2021-05-31

**Authors:** Daniel Santamaría-Aguilar, Laura P. Lagomarsino

**Affiliations:** 1 Shirley C. Tucker Herbarium, Department of Biological Sciences, Louisiana State, University, Baton Rouge, LA 70803-1705, USA Louisiana State University Louisiana United States of America; 2 Missouri Botanical Garden St. Louis, MO 63166, USA Missouri Botanical Garden St. Louis St. Louis United States of America

**Keywords:** Antioquia, Chocó, Magnoliales, Neotropics, Parque Nacional Natural Las Orquídeas, taxonomy

## Abstract

*Otoba* is the third largest genus of Myristicaceae in the Neotropics with 12 species, nine of them native to Colombia. Two new species from the department of Antioquia, *O.scottmorii***sp. nov.** and *O.squamosa***sp. nov.**, are described and illustrated. *Otobascottmorii* occurs in humid, lowland forests, while *O.squamosa* occurs in premontane forest. Previously, *Otobascottmorii* was confused with *O.acuminata* (which here is considered restricted to Costa Rica and Panama), while *O.squamosa* was confused with *O.gordoniifolia*. The similarities and differences between these and other species are discussed.

## Introduction

*Otoba* (A. DC.) H. Karst. is one of six genera of Myristicaceae native to the Neotropics. Morphologically, the genus is relatively easy to recognize. Its species have sessile or short-stalked (e.g. *O.gordoniifolia* (A. DC.) A.H. Gentry; *W. Devia et al. 2291*, MO) malpighiaceous foliar trichomes; conduplicate vernation; staminate flowers with filaments fused in an elongated column (except *O.novogranatensis* Moldenke in South America) with fused or free anthers; globose to ellipsoid green fruits; and seeds usually covered by a white aril and marked by the presence of a lateral or apical gibba (Kühn and Kubitzki 1993; Jaramillo-Vivanco and Balslev 2020). As currently delimited, the majority of *Otoba* species occur in the Andes of Colombia and Ecuador and the Chocó biogeographic region of Colombia and Ecuador, with eight and six species respectively. Outside these regions in South America, Peru, Bolivia, Venezuela, and Brazil are each home to two species; in Venezuela, these are *O.novogranatensis* and the widespread *O.glycycarpa* (Ducke) W.A. Rodrigues & T.S. Jaram., while in the remaining countries, *O.glycycarpa* and *O.parviflora* (Markgr.) A.H. Gentry can be found.

**Figure 1.**
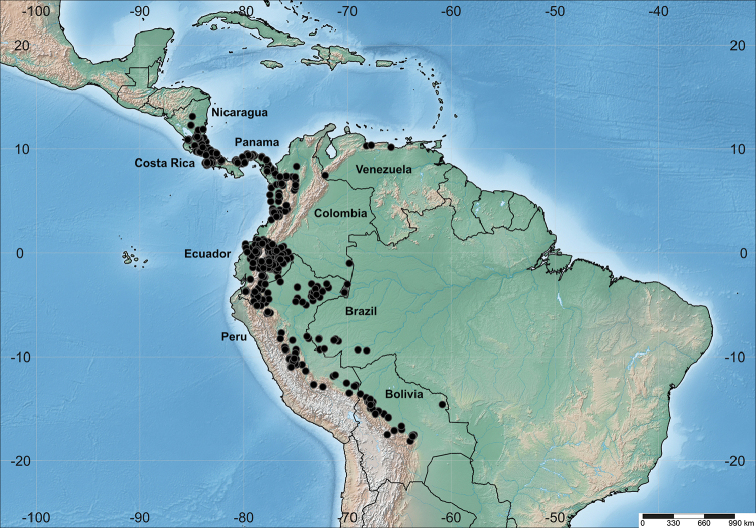
Geographic distribution of *Otoba*.

*Otoba* has been comprehensively treated twice in the last century. In the first treatment (Smith and Wodehouse 1938), in which *Otoba* was treated as *Dialyanthera* Warb., six species were recognized. Eight decades later, this was followed by a comprehensive revision of the genus now recognized as *Otoba* (Jaramillo-Vivanco and Balslev 2020). This revision recognizes 10 species distributed from Nicaragua to Brazil (Fig. 1), with six found in Central America^†^ and nine in South America. Based on morphology, species of *Otoba* can be placed into two informal groups that can be differentiated according to the anther size (0.2–0.6 vs. 0.7–1.5 mm long), vestiture on the ovary (glabrous vs. pubescent), fruits size, and pericarp thickness (see Table 1). These groups do not correspond to clades (Frost et al. 2021).

**Table 1.** Species of *Otoba* showing anther long, vestiture on the ovary, fruit size, pericarp thickness, and distributions.

Taxon	Anther long (mm)	Ovary vestiture	Fruit size (cm)	Pericarp thickness (mm)	Distribution
*O.acuminata* (Standl.) A.H. Gentry (1979: 417)	0.45–0.8	Pubescent	1.9–2.5 (−2.8) × 1.7–2.2 (−2.4)	1–2.1	Costa Rica, Panama
*O.cyclobasis* T.S. Jaram. & Balslev (2001: 563) ^‡^	*ca.* 0.3	Glabrous	1.5–2.5 × 1.5–2	1–2	Ecuador
*O.glycycarpa* (Ducke) W.A. Rodrigues & T.S. Jaram. (2001: 446) ^‡^	0.5–0.6	Pubescent	3.4 × 3.4	5–8	Colombia, Ecuador, Peru, Bolivia, Brazil
*O.gordoniifolia* (DC.) A.H. Gentry (1979: 417)^‡^	0.7–1.5	Pubescent	(3–) 5 × 4	3–5	Colombia, Ecuador
*O.gracilipes* (A.C. Sm.) A.H. Gentry (1979: 417)^‡^	0.6–0.8	Glabrous	3–3.5 × 2.5	3–4	Colombia, Ecuador
*O.latialata* (Pittier) A.H. Gentry (1979: 417)	0.2–0.3 (in Panama; pers. obs.)	Glabrous	2.2–2.8 × 1.8–2.3 (in Panama pers. obs.)	2^‡^ (ca. 1 in Panama pers. obs.)	Panama, Colombia
*O.lehmannii* (A.C. Sm.) A.H. Gentry (1979: 417)^‡^	0.7–1.3	Glabrous	4–6 × 4–4.5	3–5	Colombia
*O.novogranatensis*Moldenke (1932: 156) s.l.	1–1.5^‡^ (0.6–1.1 in Central America; pers. obs.)	Pubescent in South America^‡^; (always glabrous in Central America; pers. obs.)	3–5.5 × 2.5–4^‡^ (2.3–3.5 × 2–2.5 in Central America; pers. obs.)	2–5^‡^ ([1.4–] 2.1–2.3 in Central America; pers. obs.)	Nicaragua, Costa Rica, Panama, Colombia, Ecuador, Venezuela
*O.parvifolia* (Markgr.) A.H. Gentry (1979: 417)^‡^	0.3–0.7	Glabrous	2.3–3 × 1.5–2	1–2	Colombia, Ecuador, Peru, Bolivia, Brazil, Venezuela
*O.squamosa*	0.5–0.7	Glabrous	(2.5–) 3.4–3.6 × (1.9–) 2.8–3	2.6–3	Colombia
*O.scottmorii*	0.2–0.4	Unknown (speculated is glabrous)	1.8–2.1 × 1.5–1.7	0.68–0.89	Colombia
*O.vespertilio* D. Santam. & J.E. Jiménez (2019: 371)	ca. 0.2–0.3	Glabrous	2.2–2.7 × 1.6–1.7	1.3–1.8	Costa Rica, Panama

†*Otobagracilipes* is attributed to Costa Rica and Panama, and *O.latialata* to Costa Rica by Jaramillo-Vivanco and Balslev 2020. Part of the material that is cited as *O.gracilipes* by Jaramillo-Vivanco and Balslev (2020) corresponds to our concept of *O.acuminata* (i.e., [L.] Poveda et al. 3793, CR, MO; [P.H.] Allen 2004, MO; [T.B.] Croat 25225, MO, PMA; as well M.H. Grayum et al. 7675 CR, F [n.v.], MO, cited under *O.novogranatensis*). Other collections referred to as *O.gracilipes* in that work (i.e., [R.] Lent 2526, F digital image; [G.] Hartshorn 1310, F-2 sheets, digital imagen), as well as those referred to *O.latialata* from Costa Rica ([P.H.] Allen 5591, F digital image, CR, US digital image), and [M.H.] Grayum & [J.F.] Morales 20786 [the name of the collector correctly given to B. Hammel et al. 20786, CR-2 sheets, MO]) we consider to belong to a very broad sense of *O.novogranatensis* (D. Santam. In prep.). ‡From Jaramillo-Vivanco and Balslev 2020.

Two new species of *Otoba* are here described. Both occur in the Antioquia department of Colombia in premontane (1330–1450 m elevation) and humid (20–80 and 410–730 m) forests. Currently, there are seven species registered in this department: *O.acuminata*, *O.gordoniifolia*, *O.gracilipes*, *O.latialata*, *O.lehmannii*, *O.novogranatensis*, and *O.parvifolia* (Cogollo 2011). However, according to our interpretation, the name *O.acuminata* attributed in herbaria and literature (e.g., Cogollo et al. 2007; Jaramillo-Vivanco and Balslev 2020) has been misapplied to Colombian specimens. Instead, this name should be applied to populations from Costa Rica and Panama (see description of *O.scottmorii* below). Thus, with the inclusion of *Otobascottmorii* and *O.squamosa*, there are now eight known species of the genus for this department, and nine for the country. This makes Colombia the country with the highest number of native *Otoba* species (Table 1).

### Natural history

*Otoba* often is abundant within their habitats (Hartshorn 1983; Gentry 1993; Macía and Svenning 2005; Guevara Andino et al. 2017; Honorio Coronado et al. 2019). Illustrating this abundance, it is often one of the most collected genera in Gentry plots (i.e., those in which all plants with stem diameters equal to or exceeding 2.5 cm diameter at breast height along ten 2 × 50 m transects are documented) (Phillips and Miller 2002).

Despite its abundance, our knowledge of floral biology of *Otoba* is very limited. The small flowers, which usually have a greenish-yellow or yellow perianth, are reported to have unpleasant odors (e.g., H. van der Werff 10764, MO), sometimes reported to smell of human semen (M.H. Grayum et al. 5502, MO). Small coleopterans are known pollen vectors for *O.novogranatensis* (Flores 2010), while flowers of *O.gordoniifolia* are visited by Coleoptera (Curculionidae, Nitidulidae, Tenebrionidae), Diptera (Ceratopogonidae, Drosophilidae, Sciaridae), and Hymenoptera (Kanstrup and Olesen 2000). It is likely that all members of the genus are generalist insect pollinated, with a potentially important role for beetles, as observed in other Myristicaceae, including *Gymnacranthera* (A. DC.) Warb., *Knema* Lour., *Myristicafragrans* Houtt., and *M.insipida* R. Br. (Armstrong and Drummond 1986; Armstrong and Irvine1989; Armstrong 1997; Momose et al. 1998).

**Figure 2.**
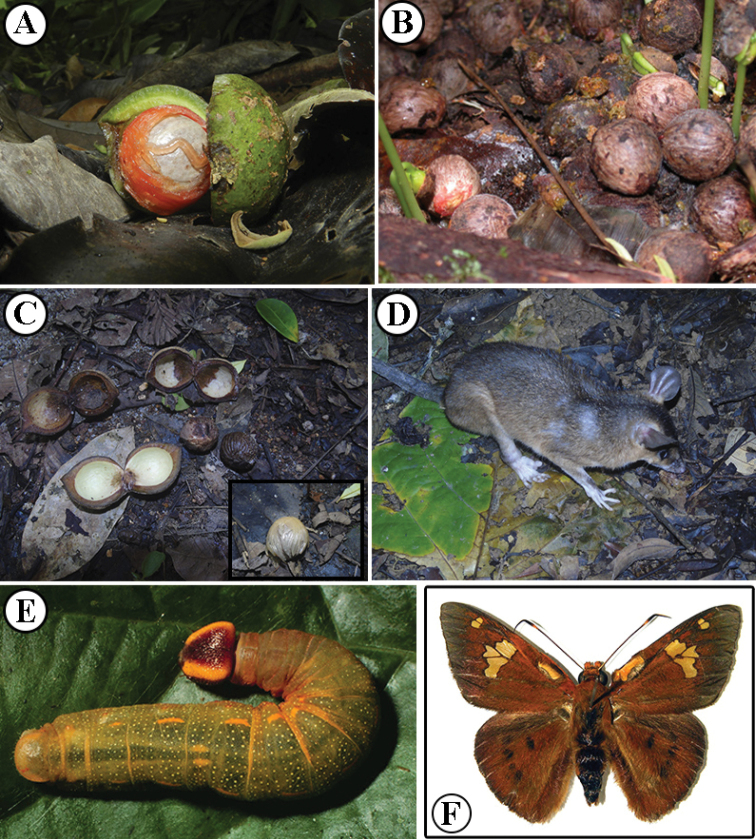
Examples of natural history of *Otoba* in Central America **A, B***Otobanovogranatensis*, fruits (**A**) and seeds (**B**) accumulating on the ground in Costa Rica **C, D** Fruits of *O.acuminata*, inset seed covered by aril (**C**), which are eaten by *Metachirusnudicaudatus* (**D**) in Panama **E, F***Dyscophellusphraxanor* (Hesperiidae), a herbivore of *O.novogranatensis*, as a larvae (**E**) and adult (**F**). Photos by Reinaldo Aguilar (**A**), Orlando Vargas (**B**) from https://sura.ots.ac.cr/florula4/index.php, Ángel Sosa-Bartuano (**C, D**), Daniel H. Janzen (**E, F**) from http://janzen.sas.upenn.edu/caterpillars/database.lasso.

More is known about dispersal in *Otoba*. Fruits are generally aromatic and globose, with a green, often shiny pericarp and a seed covered by a white or, occasionally, reddish aril. These arils can be very sweet (van Roosmalen et al. 1996; R. Aguilar, I. Chacón pers. comm.). Species with smaller fruits (~1.5–2.8 × 1.5–2.4 cm) and thin pericarps (~0.68–2.3 mm) (e.g., *O.cyclobasis*, *O.scottmorii*, Costa Rican *O.novogranatensis*, and *O.parvifolia*) are generally found at low elevations, where they are frequently reported to be dispersed by bats (Swamy 2008; Melo et al. 2009). On the other end of the fruit size spectrum, species that have large fruits (~3.4–6 × 2.8–4.5 cm) and thick pericarp (2.6–5 mm), such as *O.gordoniifolia*, *O.lehmannii*, *O.novogranatensis*, and *O.squamosa*, are likely dispersed by larger animals (birds or primates). In some of these higher elevation species, fruits are eaten by toucans (*Aulacorhynchusprasinus* in Costa Rica, *Pteroglossuserythropygius* in Ecuador) (Berg 2001; Chaves-Campos 2004) and monkeys (e.g. *Atelesbelzebuth*, *Logothrixlagotricha* in Yasuní National Park, Ecuador; *Alouattaseniculus* in Andean forests of Colombia) (Dew 2005; Giraldo et al. 2007). Various small, terrestrial animals also consume fruits of *Otoba.* This has been noted when large numbers of fallen fruit (i.e., intact pericarps and seeds; Fig. 2A, B) accumulate around the base of a tree. In Costa Rica, the red-orange arils of *O.novogranatensis* are eaten by agoutis (*Dasyproctapunctata*), pacas (*Agoutipaca*), and squirrels (pers. comm. by R. Aguilar, J.M. Chaves, F. Oviedo, J.A. Rosales). Similarly, in Panama, the white-transparent aril of *O.acuminata* is consumed by the brown four-eyed opossum, *Metachirusnudicaudatus* (Fig. 2C, D) (Sosa-Bartuano 2016). It is not known if these animals are dispersers, seed predators, or both. Germination of the seeds of *O.novogranatensis* in natural conditions is very low due to granivory by insects including a weevil (*Conotrachelus* sp., Curculionidae), although seeds collected shortly after falling germinate at high rates in nursery conditions (pers. comm. by J.A. Rosales).

Leaves of *Otoba* are also an important food source for animals. This includes mammals, such as the Caquetá titi (*Plecturocebuscaquetensis*), which eats the young leaves of *Otobaparvifolia* (Acero-Murcia et al. 2018), and herbivorous insects. In Área de Conservación Guanacaste in Costa Rica, the leaves of *O.novogranatensis* are eaten by the larvae of nine families of Lepidoptera, including *Dyscophellus* (Hesperiidae) (Fig. 2E, F) (Janzen and Hallwachs 2009).

## Materials and methods

In the course of herbarium investigations, we identified two new taxonomic species that we describe here. Descriptions are based on herbarium specimens observed at the following herbaria: CR (including ex INB), LSCR, LSU, MO, NO and USJ, as well as loaned material from: GH, NY, and US (acronyms follow Thiers 2020 [continuously updated]), though specimens from MO represent the majority of the material studied. All specimens hosted by virtual herbaria, including types specimens, were consulted, including those maintained by: the Field Museum (F; http://emuweb.fieldmuseum.org/botany/taxonomic.php), Instituto Nacional de Pesquisas da Amazônia (INPA; http://inct.florabrasil.net/en/), JSTOR Global Plants (http://plants.jstor.org), Museum of Natural History, Paris (P; http://www.mnhn.fr), and Universidad Nacional Autónoma de México (MEXU; https://datosabiertos.unam.mx/biodiversidad/). Images of specimens not available online were provided by F and JAUM. If necessary and material permitted, flowers from specimens were rehydrated before measurement. A ruler was used to measure leaves and inflorescences; a digital Neiko caliper was used to measure fruits, seeds, and the thickness of the twigs, petioles and peduncles; and, finally, flowers, trichomes and thickness of the pericarp were measured with a micrometer calibration tool (1div = 1mm) under a dissecting stereoscope (Bausch & Lomb).

In the nomenclatural section for each new species, we cite both accession numbers and barcodes when present. Barcodes are included in square brackets and follow the format of a series of numbers preceded by the herbarium acronym (e.g., [04206141]); all other numbers correspond to accession numbers.

When the coordinates and/or elevation were not included on the herbarium label but were present in the TROPICOS database (Tropicos.org. 2020), the values from TROPICOS are included in brackets. Dot-distribution maps were compiled from studied specimens and generated with SimpleMappr (Shorthouse 2010).

The preliminary conservation status of each new species was assessed using quantitative criteria recommended by the IUCN Red List (IUCN Standards and Petitions Subcommittee 2014). Georeferenced specimen data were used to determine the area of occupancy (AOO) and the extent of occurrence (EOO), which in turn were used to determine threat status. All analyses were performed in the R package conR (Dauby et al. 2017). When the recommendation differed between AOO and EOO assessments for a given species, we opted to conservatively recommend the more vulnerable status, following Knapp (2013).

## Taxonomy

### Otoba scottmorii

Taxon classificationPlantaeMagnolialesMyristicaceae

D. Santam. sp. nov.

B510B805-C140-5B10-81A1-B04ACA258316

urn:lsid:ipni.org:names:77217289-1

Figs 3 , 5B , 6C

#### Type.

Colombia. Antioquia: [Municipio] Mpio. Segovia, 24.5 km N of Remedios (17 km N of La Cruzada) on road to Zaragoza, hills side forest above río Poconé, 07°12'N, 074°48'W, [not elev.], 20 Jul. 1987 (♂ fl), W.W. Thomas & C.J. Castaño 5501 (holotype: NY! [04206141]; isotypes: INPA-159329 [digital image!], UPCB-35245 [n.v.]).

#### Diagnosis.

*Otobascottmorii* is similar to *O.cyclobasis* from Ecuador. However, it differs in having smaller leaves (5.5–8.5 [–11.2] vs. 9–14 [–18] cm long) with an attenuate to acuminate apex (vs. cuspidate), inconspicuous marginal and secondary veins (vs. conspicuous), fewer lateral veins (4–7 vs. 14–17), straight axes of staminate inflorescences (vs. zig zag), a longer filament column ([1.3–] 1.5–1.6 vs. 1 mm long), larger fruits (1.8–2.1 vs. 1.5–2.5 mm long) with thinner pericarp (0.68–0.89 vs. 1–2 mm thick), and seeds that are gibbose at the apex (vs. near-basal and lateral gibba).

#### Description.

***Tree*** (2–) 15–20 m tall × 45 cm diam., external and internal bark not described. ***Exudate*** once described as red in the bark, transparent and without specifying from where, or without exudate. ***Twigs*** 0.88–0.1 cm thick, terete to slightly flattened laterally, the external bark brown to grayish, with malpighiaceous trichomes that are 0.2–0.3 mm long, brown to ferruginous or sometimes glabrescent. ***Young foliar bud*** 0.6–1.2 cm long, densely pubescent. ***Leaves***: petiole 0.5–1.1 × 0.055–0.1 cm, canaliculate, not winged; lamina 5.5–8.5 (–11.2) × 1.7–2.5 (–3.7) cm, elliptic; adaxial side glabrous, drying blackish, sometimes brown in young leaves, the surface muricate; abaxial side drying brown to whitish grayish, the surface wrinkled to muricate, sparsely pubescent to glabrescent, with malpighiaceous trichomes that are 0.2–0.4 mm long, sessile, and ferruginous, and squamate trichomes that are ca. 0.08 mm diam. with the central portion dark and sides lighter (sometimes these sides appearing absent), with whitish crystals; vernation line absent; midvein flat to very slightly ribbed on adaxial side, the same color as the surface, abaxially 0.2–0.4 mm wide, slightly raised, darker than the surface; secondary veins brochidodromous, the loops 0.1–0.3 cm from the margin (sometimes the loops not visible), lateral veins 4–7 per side, 3–5 veins per 3 cm, on adaxial side flat and visible, on abaxial side flat and not very conspicuous, arcuate distally, the marginal vein not visible on adaxial side, slightly visible on abaxial side; tertiary veins indistinct; base attenuate, not revolute; margin entire, not revolute; apex attenuate to acuminate, the acumen 0.6–1 (–2) cm long. ***Staminate inflorescence***: axillary (only very young twigs) or supraaxillary, with 1–2 (–3) main axes, spiciform, these axes 1.5–4.3 cm × 0.41–0.1 mm, pubescent, the trichomes ferruginous to coppery, each axis compound with 2–3 (–6) fascicles of flowers, each fascicle with 1–5 (–8) flowers, alternate; bracts not seen; pedicel 3–5 mm long, pubescent; bracteoles absent. ***Staminate flowers***: flower bud 1–2.5 × 0.6–0.1 mm, elliptic to lanceolate; perianth 2–2.5 mm long, yellow or orange (in fresh material), sub-membranaceous, connate by 0.5–0.7 (–1) mm; lobes 3 (4), 1.5–1.8 × 0.8–1.1 mm, without resinous punctuations or lines, pubescent outside, the trichomes ferruginous to coppery, inside glabrous, the apex acute to obtuse, without inflexed-apiculo, the edges flat or slightly turned inwards distally, without a swollen lobed ring; filament column (1.3–) 1.5–1.6 mm long, bowling pin-shaped, fleshy, connate, glabrous; anthers 3, 0.2–0.4 mm long, free, lanceolate to oblong, apex slightly incurved. ***Pistillate inflorescence*** and ***flowers***: not seen. ***Infructescence*** 2–4 cm long, with a solitary fruit; pedicel 1.2–1.6 cm long. ***Fruits*** 1.8–2.1 × 1.5–1.7 cm, green when fresh, globose, surface glabrous, colliculate-rugose, sometimes with whitish lenticels (J. Brand 682), the line of dehiscence smooth, the base obtuse, apex apiculate, the acumen 0.1–0.3 cm long; pericarp 0.68–0.89 mm thick; seed ca. 1.6 × 1.5 cm, similar in shape to the fruit, brownish (in dry material), gibbose at the apex (ca. 0.3 mm wide), the testa ca. 0.4 mm thick; aril described once as red (E. Renteria 4680), brownish to yellowish translucent in dry material, dry and membranous in texture.

**Figure 3.**
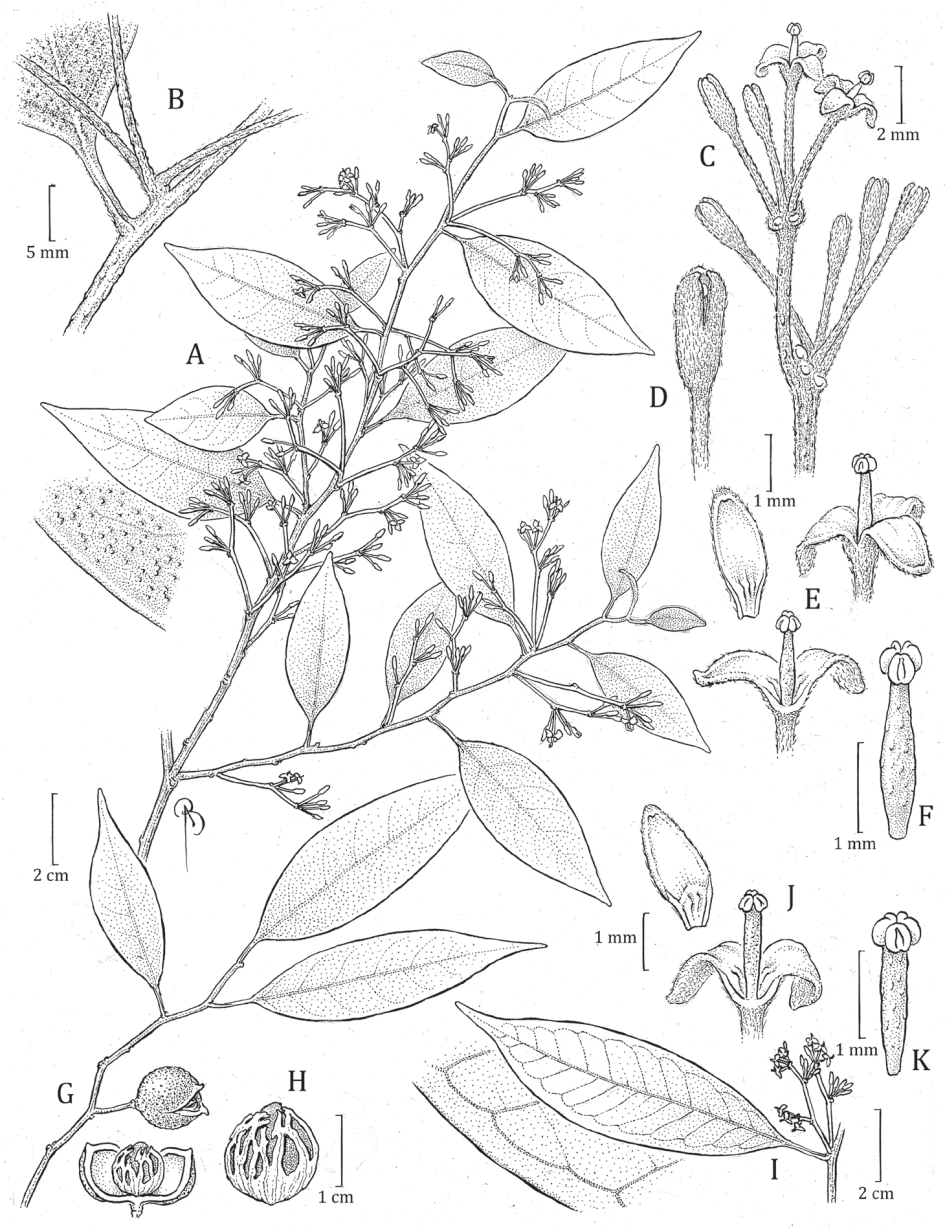
*Otobascottmorii***A** staminate flowering branch, with detail of leaf surface (detail below left) **B** leaf base, petiole and basal portion of inflorescence **C** detail of staminate inflorescence **D** flower bud **E** staminate flowers, entire (above right) and with one perianth lobe removed, showing the androecium (below center) and perianth lobe (above left) **F** androecium **G** stem with infructescence, including an immature, opening fruit **H** view of dehisced fruit (left, below G) showing the seed and aril (right) *Otobacyclobasis***I** staminate flowering branch showing leaves with brochidodromous venation, including detail of marginal vein **J** staminate flower with one perianth lobe removed, showing the androecium and perianth lobe (above left) **K** androecium. Illustration by Bobbi Angell, W.W. Thomas & C.J. Castaño 5501 (NY) (**A–F**), J. Brand & M. Narváez 682 (MO) (**G, H**), E. Narvaéz et al. 1072 (MO-2468293) (**I–K**).

#### Distinctive characters.

*Otobascottmorii* is recognized by a combination of leaf, inflorescence, and fruit traits. Its small leaf blades have long apices and thin petioles, and lack vernation lines on the abaxial surface. The staminate inflorescences are delicate with flowers on relatively thin, long pedicels; these flowers have sub-membranaceous perianth and lack a swollen-lobed ring, a bowling pin-shaped filament column, and anthers that are lanceolate to oblong. Finally, fruits are small with thin pericarp and membranous aril.

#### Etymology.

Is a pleasure to name this species after Dr. Scott A. Mori (1941–2020), a wonderful person and skilled botanist; a dedicated explorer of Central and South America humid forests (where this species occurs), especially in the Guianas and the Amazon basin; and an authority on Neotropical Lecythidaceae. His taxonomic and ecological publications gave great inspiration to the first author, as did Dr. Mori’s personal support. For an account of Dr. Mori’s legacy, see Boom (2020), Forget (2020), and Prance et al. (2021).

#### Common name.

Cuángare otobo (Cogollo et al. 2007).

#### Distribution.

**Figure 4.**
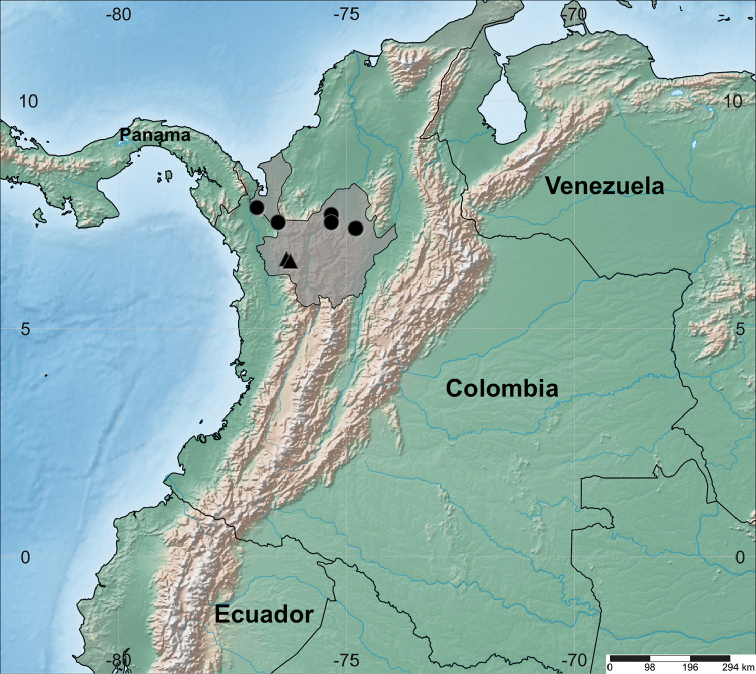
Geographic distribution of *Otobascottmorii* (black circle) and *O.squamosa* (black triangle). The shaded map area corresponds to the department of Antioquia.

*Otobascottmorii* is known only from the humid forests in the Department of Antioquia in northwestern Colombia in the Municipios of Mutatá, Segovia, Tarazá, Turbo, and Valdivia (Fig. 4). Three collections were collected under 100 m elevation (20–80 m), and three others above 400 m elevation (410–730 m).

#### Phenology.

Herbarium specimens of *Otobascottmorii* have been collected with staminate flowers in May, July, and November, and with fruits in November. Pistillate flowers have yet to be observed.

#### Preliminary conservation status.

*Otobascottmorii* is Endangered following IUCN criteria B2a. Justifying this status, it is known from five localities, has an EOO of 5,341 km^2^, and an AOO of 20 km^2^. The regions where it occurs are threatened by high levels of deforestation (González-Caro and Vásquez 2017). Of the relatively few specimens of this species that we were able to verify, the most recent specimen was collected in 1993.

#### Discussion.

**Figure 5.**
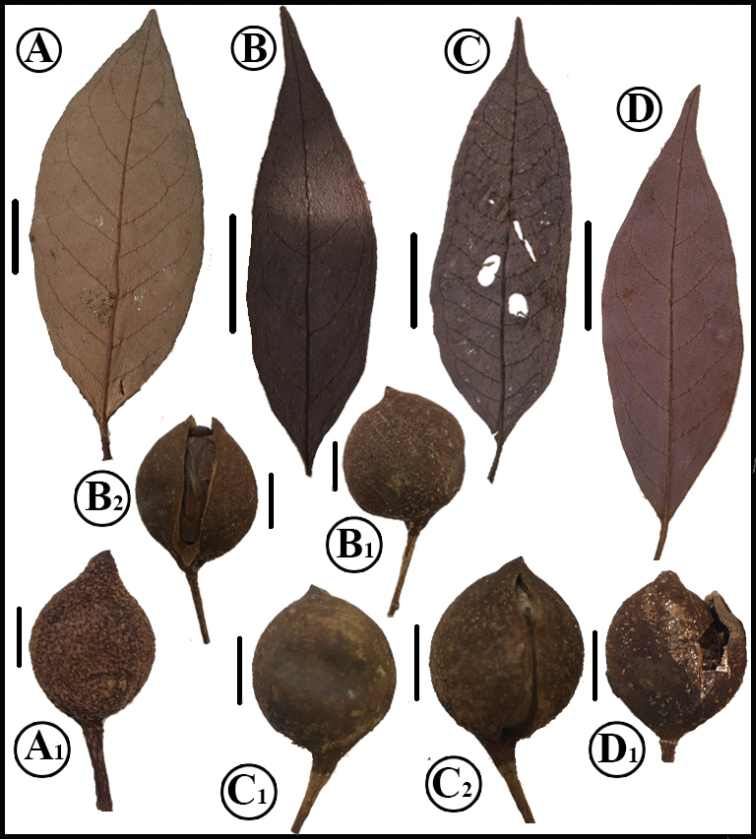
Comparison of leaf blades and fruits of *O.acuminata* (**A** L. Poveda et al. 3793, MO **A_1_** from H.W. Churchill & G.C. de Nevers 4976 MO; immature fruit) *O.scottmorii* (**B** R. Callejas et al. 3440 MO **B_1_–B_2_** from R. Callejas et al. 5789, MO) *O.cyclobasis* (**C** E. Narvaéz et al. 1072, MO **C_1_–C_2_** from W.A. Palacios & M. Tirado 11318 MO) and *O.vespertilio* (**D** G. McPherson 13597, MO, juvenile leaf, **D_1_** G. McPherson 12543, MO). Scale bars: leaves: 2 cm; fruits: 0.75 cm.

**Figure 6.**
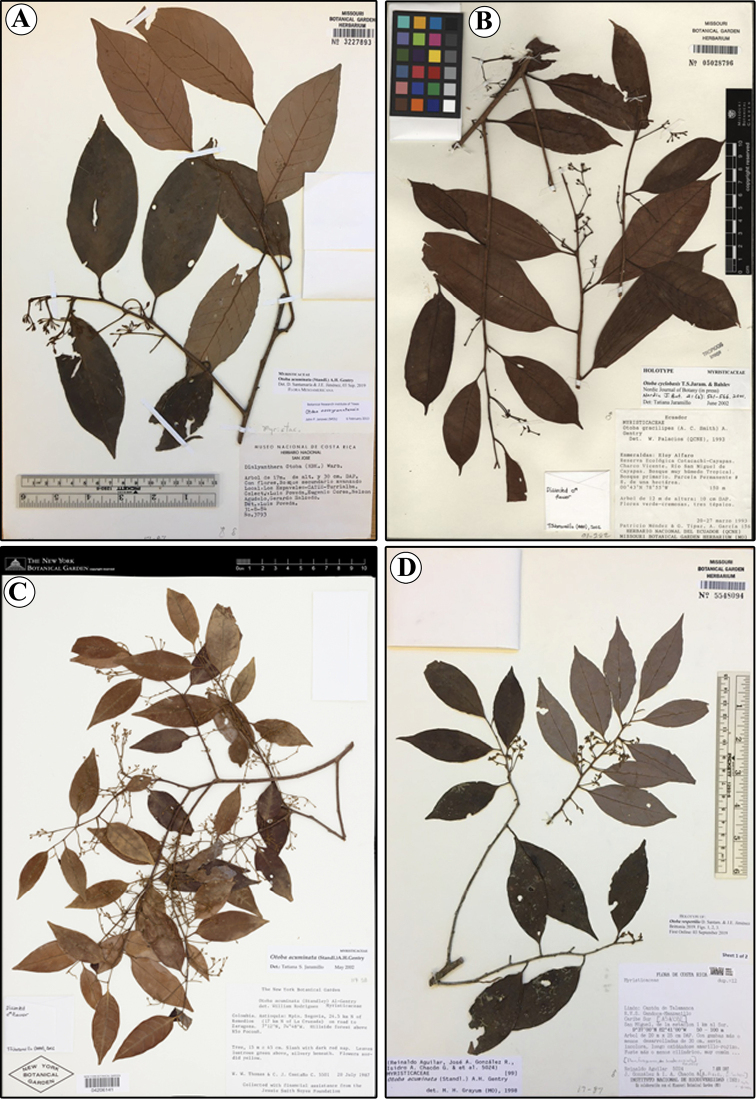
Comparations of herbarium specimens of *Otobaacuminata* (**A** L. Poveda et al. 3793, MO) *O.cyclobasis* (**B** P. Méndez et al. 156, MO) *O.scottmorii* (**C** W.W. Thomas & C.J. Castaño 5501, NY), and *O.vespertilio* (**D** R. Aguilar et al. 5024, MO).

*Otobascottmorii* is similar to *O.acuminata* and *O.vespertilio* D. Santam. & J.E. Jiménez, from Costa Rica and Panama, and *O.cyclobasis* T.S. Jaram. & Balslev from Ecuador. They all have relatively small leaf blades of similar shape and thin petioles, and usually lack vernation lines (Figs 5, 6); staminate flowers with short perianth and small anthers; and similarly sized fruit with thin pericarp (Fig. 5). The four species can be distinguished by the characteristics in Table 2.

**Table 2.** Comparison of *Otobascottmorii* with similar species (Fig. 6).

Taxon	*O.scottmorii*	*O.acuminata*	*O.cyclobasis*	*O.vespertilio*
Petiole length (cm)	0.5–1.1	(1.1–) 1.8–2.6	1–1.5^‡^	0.8–2
Leaf size (cm)	5.5–8.5 (–11.2) × 1.7–2.5 (–3.7) (Fig. 5B)	(6.5–) 8.5–15.5 × (2.7–) 4.4–6.1 (Fig. 5A)	9–14 (–18) × 3–5^‡^ (Fig. 5C)	5.3–12.5 × 2–3.5 (−4.8) (Fig. 5D)
Lateral vein number	4–7	8–12	14–17^‡^	6–8
Staminate flower pedicel length (mm)	3–5	5–8	4–5^‡^	2–3.6
Staminate perianth length (mm)	2–2.5	4.5–5.5	ca. 2.5^‡^	2–3
Perianth lobe width (mm)	0.8–1.1	1.5–2	ca. 1 ^§^	0.5–1
Ring	Absent	Absent	Present^‡^	Absent
Filament column length (mm) and shape	(1.3–) 1.5–1.6, bowling pin	2–2.7, cylindrical, ovoid, to pyriform	1, cylindrical	0.6–1.5, cylindrical
Anther length (mm) and shape	0.2–0.4 lanceolate to oblong	0.45–0.8, oblong	ca. 0.3, globose^‡^	ca. 0.2–0.3, globose to subglobose
Ovary vestiture	Unknown	Pubescent	Glabrous^‡^	Glabrous
Fruit length and width (cm)	1.8–2.1 × 1.5–1.7 (Fig. 5_1, 2_)	1.9–2.5 (−2.8) × 1.7–2.2 (−2.4) (Fig. 5A_1_)	1.5–2.5 × 1.5–2^‡^ (Fig. 5C_1, 2_)	2.2–2.7 × 1.6–1.7 (Fig. 5D_1_)
Pericarp thickness (mm)	0.68–0.89	1–2.1	1–2^‡^	1.3–1.8
Seed length and width (cm)	1.6 × 1.5	ca. 2.2 × 1.9	1–2 × 1–1.5^‡^	1.8–1.9 × ca. 1.7 cm
Distribution and elevation	Colombia, 20–80 m and 410–730 m	Costa Rica, 100–900 m; Panama, 500–1000	Ecuador, 150–300 m^‡^	Costa Rica 0–200 m, Panama 100–800 m

^‡^Information from Jaramillo-Vivanco and Balslev 2020; ^§^from E. Narvaéz et al. 1072, MO-2 sheets.

As mentioned in the introduction, *Otobascottmorii* was confused with the Mesoamerican species *O.acuminata*, which we now consider to be endemic to the Caribbean slope of Costa Rica and Panama. The specimens referred to as *O.acuminata* from Colombia in recent floras or checklists (e.g., Cogollo et al. 2007; Cogollo 2011; Ulloa Ulloa et al. 2017; Jaramillo-Vivanco and Balslev 2020) correspond to *O.scottmorii*. The confusion between these two species is likely driven by the similar morphology of the leaf blades (i.e., size and shape, usually without vernation lines, and thin petioles). However, *O.scottmorii* differs from *O.acuminata* in having narrower leaf blades (Fig. 5B, A respectively), staminate flowers with short perianth and narrow perianth lobes, a longer column of filaments, and fruits with a smooth surface and thin pericarp (Table 2). Although we have not observed the pistillate flowers of *O.scottmorii*, it is likely that the ovary is glabrous as the fruits do not have traces of trichomes, while in *O.acuminata* the ovary is pubescent; see for example: A. Rodríguez et al. 1559 (CR-2 sheets, MO); A. Estrada et al. 4829 (CR); B. Hammel & M. Grayum 14288 (CR, INPA [digital image], MEXU [digital image], MO). For a list of specimens that correspond to *O.acuminata*, see Santamaría-Aguilar et al. (2019).

#### Notes.

The type specimen of *Otobascottmorii* and other collections here mentioned have been previously identified in herbaria and cited in literature as *O.acuminata* and *O.gracilipes*, and duplicates may have been distributed under these names.

#### Specimens examined.

**Colombia. Antioquia**: Mutatá, Sitio Rio Surambay, 12 km N de Mutata, 07°20'N, 076°30'W, 30–80 m, 21 Nov. 1987 (♂ fl), R. Callejas et al. 5752 (INPA digital image, MO, NY); ibid, 21 Nov. 1987 (fr), R. Callejas et al. 5789 (INPA digital image, MO, NY); Turbo, Carretera Tapón del Darién, Sector Río León-Lomas Aisladas, km 37, [07°39'11"N, 076°58'02"W], 20 m, 29 Nov. 1993 (fr), J. Brand & M. Narváez 682 (COL [n.v.], JAUM digital image, MO); río Cianurá, Paso de la Reina, 730 m, 13 Mar 1986 (fr), E. Renteria et al. 4680 (JAUM digital image); Tarazá, Corregimiento El 12, 210 kms, NE de Medellín, vía El 12-Barroblanco, km 3, 07°30'N, 075°20'W, [450 m], 09 Nov. 1987 (♂ fl), R. Callejas et al. 5509 (INPA digital image, NY); Valdivia, corregimiento Puerto Valdivia, km 5 de Puerto Valdivia hacia “El 12”, colecciones a lo largo del Río Pescado, 07°20'N, 075°20'W, 410 m, 14 May. 1987 (♂ fl), R. Callejas et al. 3440 (MO-2 sheets, NY).

### Otoba squamosa

Taxon classificationPlantaeMagnolialesMyristicaceae

D. Santam. sp. nov.

95FD5676-9981-59B1-A17D-BF9200F2466E

urn:lsid:ipni.org:names:77217290-1

Fig. 7

#### Type.

Colombia. Antioquia: Urrao, Vereda Calles, Parque Nacional Natural “Las Orquídeas”, margen derecha Quebrada La Honda, 06°32'N, 076°19'W, 1330–1400 m, 08 May 1993 (fr), Á. Cogollo, R. Carmona, E. Álvarez 6197 (holotype: MO-05011088! [1500541]; isotypes: n.v.).

#### Diagnosis.

*Otobasquamosa* is similar to *O.gordoniifolia* from Colombia and Ecuador, and both species grow in montane forest in the Andes. However, it differs in its leaves with shorter petioles (1.7–2.7 [–3.8] vs. [3–] 5–7 cm long) and smaller lamina (6.7–14.5 vs. [13–] 24–34 cm long), staminate flowers with a perianth with a swollen ring (vs. without) and smaller anthers (0.5–0.7 vs. 0.7–1.5 mm long), pistillate flowers with a glabrous ovary (vs. pubescent), and fruits with thin pericarp (2.6–3 vs. 3–5 mm thick).

**Figure 7.**
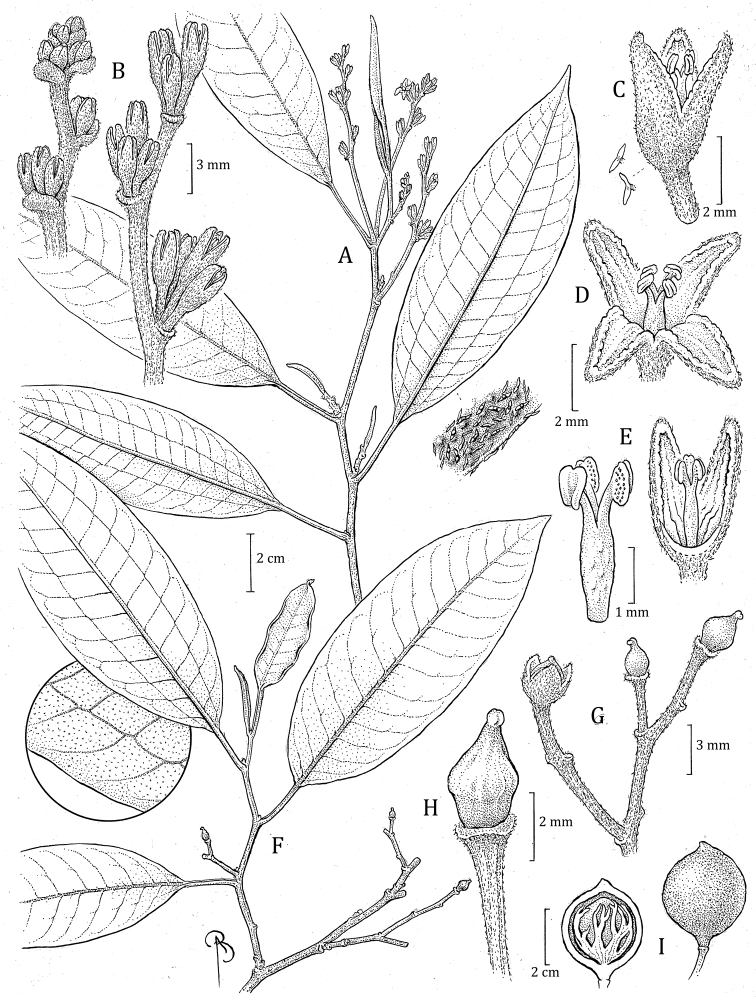
*Otobasquamosa***A** staminate flowering branch, with detail of malpighiaceous trichomes on abaxial leaf surface (detail below right) **B** detail of staminate inflorescence **C** staminate flower, with detail of trichomes **D** open staminate flower **E** longitudinal section of staminate flower and androecium **F** pistillate flowering branch **G** portion of pistillate inflorescence, showing the perianth (left) and ovary after the perianth has fallen **H** ovary **I** closed and open fruit (left), showing the pericarp and aril. Illustration by Bobbi Angell. Á. Cogollo et al. 6279 (MO) (**A–D**), J. Pipoly et al. 17019 (MO) (**E**), J. Pipoly et al. 16798 (**G**), Á. Cogollo et al. 6197 (MO) (**F, H, E**).

#### Description.

***Tree*** 8–18 (–24) m tall × 11.9–34.7 cm diam., external and internal bark not described. ***Exudate*** hyaline, oxidizing reddish, only reported from in flowers and fruits. ***Twigs*** 0.13–0.27 cm thick, terete to slightly flattened laterally, the external bark brown to blackish, with malpighiaceous trichomes 0.2–0.6 mm long, brown to ferruginous, the indument denser in young parts. ***Young foliar bud*** 1.6–4.3 (–5.8) cm long, densely pubescent. ***Leaves***: petiole 1.7–2.7 (–3.8) × 0.1–0.2 (–0.25) cm, canaliculate, very short-winged; lamina 6.7–14.5 × 3.4–6 (–8) cm, elliptic, rarely widely elliptic; adaxial side glabrous, usually drying dark brown to blackish, the surface muricate-reticulate; abaxial side usually drying pale to dark brown, the surface muricate, sparsely pubescent, with malpighiaceous trichomes 0.3–0.6 mm long, sessile, ferruginous, and scale-like trichomes ca. 0.1 diam. with the central part dark, contrasting with the lighter sides, crystals generally absent and if present very few; vernation line imprints 2 parallel lines, 0.7–1.5 (–1.8) cm from the margin, the panel area 1.8–2.5 (–3.4) cm wide (in the central portion), the same color as the surface; midvein flat on adaxial side, the same color as the surface or blackish, abaxially 0.5–0.9 mm wide, raised, a little darker than the surface; secondary veins brochidodromous, the loops 0.2–0.3 cm from the margin, lateral veins 13–17 per side, (3–) 4–6 veins per 3 cm, on adaxial side slightly caniculate, on abaxial side flat to slightly raised, not very conspicuous, arcuate distally, the marginal vein not visible on adaxial side, slightly visible on abaxial side; tertiary veins indistinct; base acute to cuneate, not revolute; margin entire, not revolute; apex acute, the acumen 0.3–0.9 cm long. ***Staminate inflorescence***: axillary and/or ramiflorus, with 1–2 main axes, spiciform, these axes 2–7 cm × 0.6–1.2 mm, pubescent, the trichomes ferruginous, each axis compound with 2–5 fascicles of flowers, each fascicle with 3–6 flowers, alternate; bracts ca. 1.5–1.6 × 1 mm (observed in very young inflorescences), densely pubescent outside, the trichomes ferruginous; pedicel 1.3–4 mm long, pubescent; bracteoles absent. ***Staminate flowers***: flower bud 2–3 × 1–1.5 mm; perianth 3.5–4.7 mm long, yellowish to yellowish-green (in fresh material), fleshy (hardening near the base by the ring), connate by 1–1.7 mm; lobes 3 (4), (2–) 2.5–3.4 × (0.9–) 1.4–2 mm, without resinous punctuations or lines, pubescent outside, the trichomes ferruginous, inside glabrous, smooth to lightly spongy, the apex in some flowers with a minutely inflexed-apiculate, the margin edges slightly turned inwards, slightly wavy; ring present, 0.1–0.4 mm wide, lobed, smooth, or sometimes spongy; filament column 1.5–2.1 mm long, usually cylindrical, slightly narrow towards the apex, fleshy, glabrous; anthers 3 (4), 0.5–0.7 mm long, free, lanceolate to oblong, apex slightly incurved. ***Pistillate inflorescence***: axillary, 1.4–3.2 cm long, pubescent, the trichomes ferruginous, each axis compound with 1–2 fascicles of flowers, each fascicle with 2–3 flowers, alternate; bracts not seen; pedicel 3–4 mm long, pubescent; bracteoles absent. ***Pistillate flowers***: flower bud 3–4 × ca. 2 mm; perianth 3–5 mm long, the color and texture as in the staminate flower, connate by 1–1.5 mm; lobes 3 (4), 2–3.5 × 2–2.5 mm, without resinous punctuations or lines, pubescent outside, the trichomes ferruginous, inside glabrous; ring present; gynoecium 2–3 × 1.5–2.3 mm, glabrous, ovary sessile to short-stalked, ca. 0.6 mm long; stigma 2-lipped, subsessile; stigmatic lips ca. 0.6 mm long. ***Infructescence*** probably with one fruit (fruits separated from the axis in all specimens observed); pedicel ca. 1–1.1 cm long. ***Fruits*** (2.5–) 3.4–3.6 × (1.9–) 2.8–3 cm, green, globose, the surface glabrous, rugose, sometimes with whitish to brownish lenticels, the line of dehiscence smooth, the base obtuse, and sometimes getting narrower towards the pedicel, apex obtuse or acute, the acumen 0.8–1 cm long; pericarp 2.6–3 mm thick; seed (2.1–) 2.5–2.9 × (1.7–) 2.3–2.4 cm, similar in shape to the fruit, whitish or brown (in dry material), gibbose at the apex or nearly so, the testa 0.4–0.6 mm thick; aril described once as white (D. Sánchez et al. 1529), brownish to white-yellowish in dry material, waxy to dry in texture.

#### Distinctive characters.

*Otobasquamosa* is recognized by a variety of leaf traits, including: squamate or scale-like indument mixed with malpighiaceous trichomes on the abaxial surface, lateral veins that are more or less conspicuous, forming a marginal vein, and vernation lines that parallel the midvein. Additionally, the staminate flowers have a perianth with swollen-lobed ring in the inner surface, a typically cylindrical filament column with lanceolate to oblong anthers and pistillate flowers that have a glabrous gynoecium. Finally, the fruits are relatively large, with thick pericarp.

#### Etymology.

The specific epithet refers to the squamate or scale-like indument that is present with more typical malpighiaceous trichomes on the abaxial surface of the leaf blades. The squamate or scale-like indument is not unique to this species; it is also present in most specimens of *O.acuminata*, *O.glycycarpa*, *O.scottmorii*, and *O.vespertilio*; also Smith and Wodehouse (1938), they described for *O.lehmannii* (as *Dialyanthera Lehmannii*).

#### Common name.

None recorded.

#### Distribution.

*Otobasquamosa* is known from the Cordillera Occidental of Colombia, specifically in the municipalities of Frontino and Urrao in the Department of Antioquia (Fig. 4). It grows in premontane forest between 1330–1450 m.

#### Phenology.

Fertile herbarium specimens of *Otobasquamosa* have been collected with staminate flowers in May and December, with pistillate flowers in May, September, and December, and with fruits in March, May, September, October, and December.

#### Preliminary conservation status.

*Otobasquamosa* is Endangered following IUCN criteria B2a. Justifying this status, it is known from only two localities and has an AOO of 4 km^2^; there are too few verified localities to reasonably estimate the EOO. The Andean forests of the Antioquia Department of Colombia where it occurs are particularly at risk for deforestation (González-Caro and Vásquez 2017).

#### Discussion.

**Figure 8.**
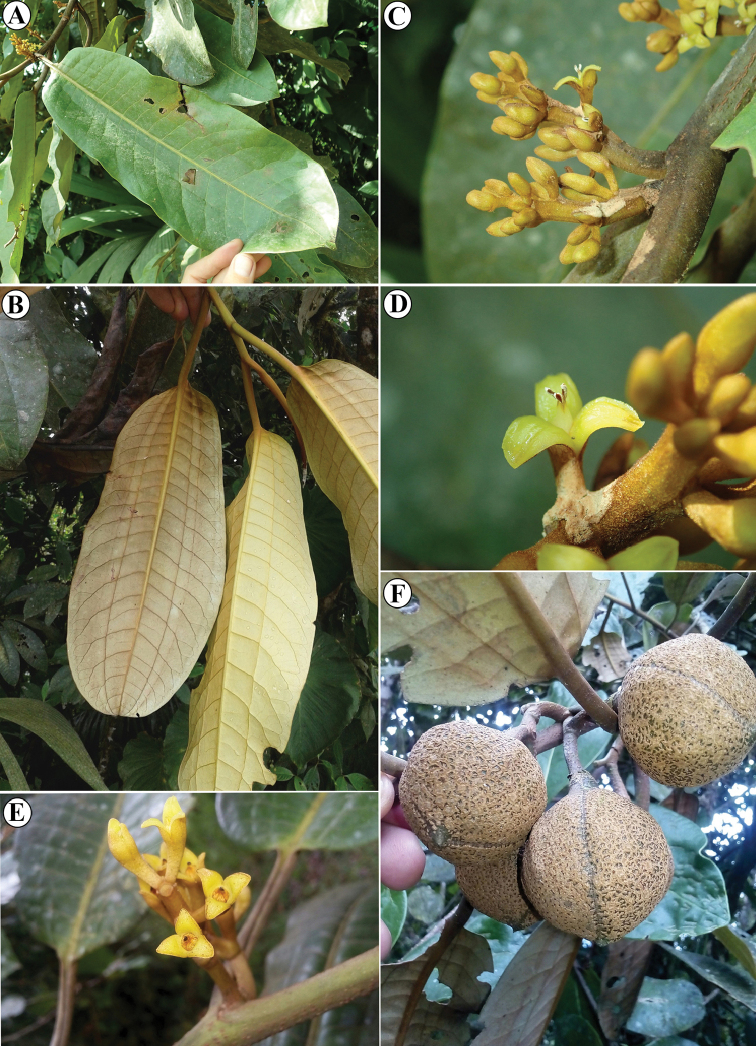
*Otobagordoniifolia***A** leaf on adaxial side **B** leaf on abaxial side, illustrating long petioles and vernation lines **C** staminate inflorescence **D** staminate flower **E** pistillate flowers, note the pubescent ovary **F** immature fruits. Photos by Rudy Gelis taken in Ecuador (unvouchered).

The collections now identified as *Otobasquamosa* were previously included under the concept of *O.gordoniifolia* (Fig. 8), and identified as such in herbarium specimens and in the literature (e.g., Cogollo 2011). These species share similarities: both grow in montane forests and have the leaf blades with conspicuous vernation lines (Figs 7A, F, 8B), long petioles (shorter in the new species), lanceolate to oblong anthers, and fruit with thick pericarp. However, *Otobasquamosa* differs in its smaller leaf blades with thinner petioles, staminate flowers with a shorter perianth with a swollen-lobed ring in the inner surface and smaller anthers, and pistillate flowers with glabrous ovaries. See Table 3 for a comparison of these distinguishing characteristics. In addition, *O.squamosa*’s leaf blades are less pubescent abaxially, the terminal young foliar bud is shorter, and petioles are shorterwinged.

**Table 3.** Comparison of *Otobagordoniifolia*, and *O.squamosa*.

	*Otobasquamosa*	*O.gordoniifolia* ^‡^
Petiole length (cm)	1.7–2.7 (–3.8) × 0.1–0.2 (–0.25)	(3–) 5–7 × 0.3–0.4
Leaf size (cm)	6.7–14.5 × 3.4–6 (–8)	(13–) 24–34 × (6–) 8–12
Staminate perianth length (mm)	3.5–4.7	(2–) 4–6
Ring	Present	Absent
Anthers length (mm)	0.5–0.7	0.7–1.5
Ovary vestiture	Glabrous	Pubescent
Fruit size (cm)	(2.5–) 3.4–3.6 × (1.9–) 2.8–3	(3–) 5 × 4
Pericarp thickness (mm)	2.6–3	3–5

^‡^From Jaramillo-Vivanco and Balslev 2020

Within *Otoba*, the swollen-lobed ring in the staminate perianth is shared between *O.cyclobasis* (and even gives this species its specific epithet; Jaramillo and Balslev 2001) and *O.squamosa*; in addition to this feature, both species have glabrous ovaries. However, the new species has longer petioles (1.7–2.7 [–3.8] vs.1–1.5 cm) and leaves (6.7–14.5 vs. 3.4–6 [–8] cm), lanceolate to oblong anthers (vs. globose), and larger fruits ([2.5–] 3.4–3.6 vs. 1.5–2.5 cm long); further, *O.squamosa* grows at higher elevations (1330–1450 vs. 150–300 m).

In the “Key to the species (pistillate or fruiting plants)” from Jaramillo-Vivanco and Balslev (2020), *Otobasquamosa* matches the recently described *O.vespertilio* from Costa Rica and Panama, though it is clearly not conspecific. *Otobasquamosa* differs from *O.vespertilio* in its longer petioles (1.7–2.7 [–3.8] vs. 0.8–2 cm long), abaxially conspicuous vernation lines (vs. absent), staminate perianth with a swollen-lobed ring (vs. without), and larger fruits ([2.5–] 3.4–3.6 vs. 2.2–2.7 × 1.6–1.7 cm) with thicker pericarp (2.6–3 vs. 1.3–1.8 mm).

#### Notes.

The specimen [J.] Pipoly 17003 (MO nv), included in Jaramillo-Vivanco and Balslev (2020), was collected in the same area of the type and other material of *O.squamosa* cited here, and we suspect that it corresponds to this new species, though this specimen was not located in our study.

Specimens of *O.squamosa*, including the type, have been previously identified in herbaria as *O.gordoniifolia*, and duplicates may have been distributed under these names.

The following sterile specimens from MO were collected in the same locality as fertile material or nearby (Parque Nacional Natural “Las Orquídeas”, Sector Calles); because they were not fertile, they were not used in the description presented above, though we believe that they correspond to this new species: Á. Cogollo et al. 6069, 6132, 6169, 6298, 6344, 6642, 6644, 6657, 6683, 6684, 6869, 6926, 6929, 7109, 7180, 7221, 7237, 7238, 7259, 7262, 7268, 7269; and J. Pipoly et al. 16663, 17022, 17033, 17042, and 17076.

#### Specimens examined.

**Colombia**. **Antioquia**: Frontino, Corregimiento Nutibara, cuenca alta del Río Cuevas, bosque al lado carretera a La Blanquita, 1100 m, 21 Sep. 1987 (♀ fl & fr), D. Sánchez et al. 1529 (MO); Urrao, Parque Nacional Natural “Las Orquídeas”, Sector Calles, margen derecha del Río Calles, 06°32'N, 076°19'W, 1420 m, 25 Mar. 1988 (fr), Á. Cogollo et al. 2573 (MO); Vereda Calles, Parque Nacional Natural “Las Orquídeas”, margen derecha Quebrada La Honda, 06°32'N, 076°19'W, 1330–1400 m, 03 May 1993 (♂ fl), Á. Cogollo et al. 6074 (MO); ibid, 08 May 1993 (♂ fl), Á. Cogollo et al. 6190 (K-2 sheets [n.v.], MO); ibid, 08 May 1993 (♀ fl), Á. Cogollo et al. 6198 (MO); ibid, 11 May 1993 (♂ fl), Á. Cogollo et al. 6279 (MO, NY); ibid, 11 May 1993 (♂ fl), Á. Cogollo et al. 6300 (K n.v., MO); Vereda Calles, Parque Nacional Natural “Las Orquídeas”, margen derecha del Río Calles, en el filo NW de la Cabaña de Calles, 06°32'N, 076°19'W, 1450 m, 15 Oct. 1993 (fr), Á. Cogollo et al. 6918 (MO); ibid, 18 Oct. 1993 (fr), Á. Cogollo et al. 7107 (MO); ibid, 08 Dec. 1993 (fr), Á. Cogollo et al. 7959 (GH digital image, K [n.v.], MO, NY); Las Orquídeas, Vereda Calles, Parque Nacional Natural Las Orquídeas, Quebrada Honda, 06°29'N, 076°14'W, 1300 m, 08 Dec. 1992 (fr), J. Pipoly et al. 16745 (MO); ibid, 1330 m, 08 Dec. 1992 (♀ fl), J. Pipoly et al. 16798 (MO); ibid, 10 Dec. 1992 (♂ fl), J. Pipoly et al. 16902 (MO); ibid, 1300 m, 11 Dec. 1992 (♂ fl), J. Pipoly et al. 17019 (K [n.v.], MO); Parque Nacional Natural Las Orquídeas, Vereda Calles, margen derecha del Río Calles, 06°32'N, 076°19'W, 1350–1450 m, 05 Dec. 1993 (fl bud), J. Pipoly et al. 17721 (MO).

The identification key below is modified from Jaramillo-Vivanco and Balslev 2020. Except where specified otherwise, the information comes from Smith 1950^|^, measurements of the digital image of the holotype^¶^, or measurements of herbarium specimens^#^. The key was difficult to build, in part because of current limited access to physical specimens during the COVID-19 pandemic and in part because we were not able to identify obvious characters to separate species in some cases (e.g., between *O.gordoniifolia* and *O.lehmannii*). This may mean that the key is difficult to use, but we have decided to include it in case it is of use to some.

## Key to the species of *Otoba* in Colombia

**Table:** 

1	Abaxial leaf blades with vernation lines (e.g. 7A, 8B).	
2	Leaf blades 6.7–14.5 cm long, abaxial surface with sparse, scale-like and trichomes; perianth of staminate flowers with swollen-lobed ring; staminate flowers with anthers 0.5–0.7 mm long; fruits with pericarp 2.6–3 mm thick	***O.squamosa***
2'	Leaf blades (13–) 24–43 cm long, abaxial surface with dense, scale-like and trichomes; perianth of staminate flowers without swollen-lobed ring; staminate flowers with anthers 0.7–1.5 mm; fruits with pericarp 3–5 mm thick.	
3	Pistillate flowers with pubescent ovaries	***O.gordoniifolia***
3'	Pistillate flowers with glabrous ovaries	***O.lehmannii***
1'	Abaxial leaf blades without vernation lines.	
4	Leaf blades with petioles less than 0.2 cm thick.	
5	Staminate inflorescence 6–7 cm long, flower bud 1.2–1.6 mm wide^|^, perianth ca. 3 mm long; filament column divided distally; anthers ca. 0.5 mm long	***O.gracilipes ^¶^***
5'	Staminate inflorescence 1.5–4.3 cm long, flower bud 0.6–0.1 mm wide, perianth 2–2.5 mm long; filament column completely connate; anthers 0.2–0.4 mm long	***O.scottmorii***
4'	Leaf blades with petioles up to (0.1–) 0.2 cm thick.	
6	Staminate flowers with filament column distinct almost to the base	***O.novogranatensis***
6'	Staminate flowers with filament column connate, if distinct never to the base	
7	Leaf blades with petioles conspicuously winged; staminate inflorescence with the first fascicles of flowers 2.2–4 (–8) cm^#^ from the base, fascicles of flowers arranged more or less in a zig-zag, fascicles well separated (1–1.5 cm apart^#^), pedicel filiform, flowers with membranaceous perianth	***O.latialata***
7	Leaf blades with petioles usually not winged, if winged, the wing very narrow; staminate inflorescence with the first fascicles of flowers 0.5–2 cm^#^ from the base, fascicles of flowers arranged in a straight line, each fascicle in close proximity (0.5–1 cm apart^#^), pedicel stout, flowers with fleshy perianth.	
8	Abaxial leaf surface densely covered with scale-like trichomes; pistillate flowers with pubescent gynoecium; fruits with pericarp 5–8 mm thick	***O.glycycarpa***
8'	Abaxial leaf surface sparsely covered with scale-like trichomes; pistillate flowers with glabrous gynoecium; fruits with pericarp 1–2 mm thick	***O.parvifolia***

## Supplementary Material

XML Treatment for Otoba scottmorii

XML Treatment for Otoba squamosa
